# PCSK9 Biology and Its Role in Atherothrombosis

**DOI:** 10.3390/ijms22115880

**Published:** 2021-05-30

**Authors:** Cristina Barale, Elena Melchionda, Alessandro Morotti, Isabella Russo

**Affiliations:** Department of Clinical and Biological Sciences, Turin University, I-10043 Orbassano, TO, Italy; cristina.barale@unito.it (C.B.); e.melchionda@gmail.com (E.M.); alessandro.morotti@unito.it (A.M.)

**Keywords:** proprotein convertase subtilisin/kexin type 9, hypercholesterolemia, low-density lipoprotein, low-density lipoprotein receptor, atherosclerosis, platelets, thrombosis

## Abstract

It is now about 20 years since the first case of a gain-of-function mutation involving the as-yet-unknown actor in cholesterol homeostasis, proprotein convertase subtilisin/kexin type 9 (PCSK9), was described. It was soon clear that this protein would have been of huge scientific and clinical value as a therapeutic strategy for dyslipidemia and atherosclerosis-associated cardiovascular disease (CVD) management. Indeed, PCSK9 is a serine protease belonging to the proprotein convertase family, mainly produced by the liver, and essential for metabolism of LDL particles by inhibiting LDL receptor (LDLR) recirculation to the cell surface with the consequent upregulation of LDLR-dependent LDL-C levels. Beyond its effects on LDL metabolism, several studies revealed the existence of additional roles of PCSK9 in different stages of atherosclerosis, also for its ability to target other members of the LDLR family. PCSK9 from plasma and vascular cells can contribute to the development of atherosclerotic plaque and thrombosis by promoting platelet activation, leukocyte recruitment and clot formation, also through mechanisms not related to systemic lipid changes. These results further supported the value for the potential cardiovascular benefits of therapies based on PCSK9 inhibition. Actually, the passive immunization with anti-PCSK9 antibodies, evolocumab and alirocumab, is shown to be effective in dramatically reducing the LDL-C levels and attenuating CVD. While monoclonal antibodies sequester circulating PCSK9, inclisiran, a small interfering RNA, is a new drug that inhibits PCSK9 synthesis with the important advantage, compared with PCSK9 mAbs, to preserve its pharmacodynamic effects when administrated every 6 months. Here, we will focus on the major understandings related to PCSK9, from its discovery to its role in lipoprotein metabolism, involvement in atherothrombosis and a brief excursus on approved current therapies used to inhibit its action.

## 1. Introduction

Hypercholesterolemia is unequivocally the most well-established risk factor associated with the development of atherosclerotic cardiovascular (CV) disease (CVD), which, in turn, accounts for the plurality of worldwide morbidity and mortality [1]. Disorders in cholesterolemia clearance can be primary (genetic) or secondary to other pathologies. Among the primitive forms, familial hypercholesterolemia is the most frequent among the genetic causes of early CVD because of lifelong exposure to dyslipidemia.

With the advent of statins, a sustained and effective reduction in low-density lipoprotein cholesterol (LDL-C) levels has been achieved and large-scale clinical trials show that the reduction in LDL-C is associated with a significant reduction in adverse CV events [2,3]. 

Despite the success of LDL-C lowering by statins, less than half of recurrent CV events can be prevented and, even with adjunctive lipid-lowering therapies, such as ezetimibe, certain patients do not achieve satisfactory control of dyslipidemia.

Proprotein convertase subtilisin kexin 9 (PCSK9) has lately received considerable attention as a target for the reduction of LDL-C levels in patients with hypercholesterolemia [4].

Therefore, PCSK9 inhibitors have become effective and exciting new therapies for lipid management, especially for patients who could not achieve adequate lipid-lowering with combination therapy of maximally tolerated statin and ezetimibe. 

Given that enhanced circulating levels of LDL-C are crucial not only for the development and the progression but also for the outcomes of atherosclerotic CV disease [5], therapies including PCSK9 inhibitors are expected to slow the progression of atherosclerosis and reduce CV events and death. Indeed, the pharmacological approach with PCSK9 inhibitors significantly and safely improves cardiovascular outcomes, even though a significant CV benefit in terms of mortality has not been shown yet and there being an absence of specific long-term trials [6,7,8].

The central role of PCSK9 remains the regulation of cholesterol homeostasis even if growing evidence confirms that PCSK9 can exert a plethora of pleiotropic effects [9]. In particular, PCSK9 effects on vascular biology through additional atherogenic mechanisms are known to promote inflammation, plaque formation and thrombosis, events all involved in the pathogenesis of acute coronary syndrome (ACS).

## 2. PCSK9 Discovery

Insights into the physiological function of PCSK9 (initially named neural apoptosis-regulated convertase-1) were derived initially from the finding that functional mutations in the *PCSK9* gene cause dominant familial hypercholesterolemia (ADH). A paper published in 2003 described a natural mutant of *PCSK9* discovered in French families with no mutation in the *LDLR* or *apolipoprotein B* (APOB) genes showing severely high LDL-C levels [10]. Single-point gain-of-function (GOF) mutations (p.S127R and p.F216L) in the *PCSK9* gene revealed the involvement of an as-yet-unknown actor in cholesterol homeostasis. Additional GOF variants of PCSK9 linked to high plasma LDL-C [11] confirmed its pathophysiological role and PCSK9 became in a short time a promising treatment target in the clinic. 

The interest in PCSK9 function was also strengthened by the study carried out on a multiethnic population (50% African Americans) in Dallas County, USA [12], where two nonsense mutations in *PCSK9* (p.Y142X and p.C679X) occurred at a combined frequency of ∼2% in African Americans, and were associated with 40% lower levels of LDL-C. It was subsequently demonstrated that these nonsense mutations were associated with reduced life-long LDL-C exposure and reductions in coronary artery disease risk [13].

In 2006, Zhao et al. [14] described for the first time an individual, a healthy woman, with no immunodetectable circulating PCSK9 concentrations and very low plasma levels of LDL-C (14 mg/dL) because of the compound heterozygote of the two inactivating mutations that disrupt PCSK9 synthesis (paternal allele) and secretion (maternal allele). In another study where *PCSK9* nonsense mutations were detected in African subjects, one woman was homozygous for C679X with severe hypocholesterolemia (LDL-C levels of 7 mg/dL) without apparent medical problems [15].

Since then, PCSK9 function has received such a remarkable bulk of research that it is considered a striking example of a rapid translation from a genetic-based discovery to an approved therapy.

## 3. PCSK9 Gene and Structure

PCSK9 is the ninth and last member of a family of serine proteases closer to bacterial subtilisin called proprotein convertase 1 (PC1), PC2, furin, PC4, PC5, paired basic amino acid cleaving enzyme 4 (PACE4), PC7, subtilisin kexin isozyme 1 (SKI-1; also known as S1P) and PCSK9 [16,17]. Data from in vitro and ex vivo studies as well as phenotypes associated with human mutations or knockout animal models show that enzymes of this family can exert unique physiological roles [16], and are implicated in the processing and/or modulation of various proteins that determine their activation or inactivation [18]. The first eight convertases—PC1, PC2, furin, PC4, PC5, PACE4, PC7 and SKI-1—cleave secretory protein precursors to generate mature, functional and bioactive peptides, polypeptides and hormones involved in regulating growth and metabolism [19,20]. In contrast, PCSK9 cleaves itself and then no longer functions as a protease [21], rather it acts in a non-enzymatic fashion to promote the endosomal and lysosomal degradation of the most prominent receptor involved in LDL-C homeostasis (LDLR) [22,23]. Therefore, the catalytic activity of PCSK9 is not needed for its functional enhancement on LDLR cycling.

**Figure 1:**
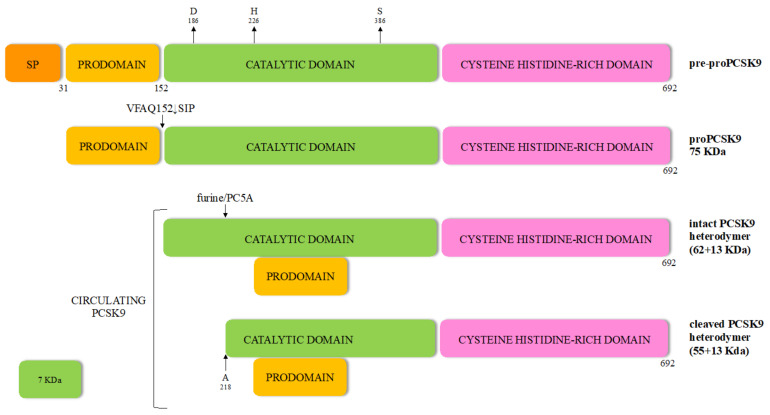
Processing of the zymogen pre-proPCSK9 leading to the cleaved PCSK9 mature form. SP: signal peptide.

The 22-kb human *PCSK9* gene is located at chromosome 1p32 and comprises 11 introns and 12 exons that encode a 72-kDa zymogen of 692-amino acids synthesized in the endoplasmic reticulum (ER) as a glycoprotein, but is still unable to bind to LDLR (Figure 1). 

The proprotein contains a signal peptide (SP) (residues 1 to 30), an N-terminal domain (prodomain, residues 31 to 152) and a catalytic domain (residues 153 to 451) with the typical catalytic triad aspartic acid, histidine and serine residues, as well as the asparagine residue comprising the oxyanion hole [23]. The carboxy-terminal domain (residues 452 to 692) contains a sequence that regulates PCSK9 cellular localization, exhibiting a cysteine, histidine-rich domain (CHRD). This specific domain reveals a structural homology to the resistin homotrimer—a small cytokine associated with obesity and diabetes-and required for the trafficking of the PCSK9-LDLR complex to endosomes and lysosomes [24,25]. Indeed, like most secretory precursor proteins, PCSK9 is subject to various post-translational modifications before becoming fully competent to bind its target. The first event occurs co-translationally in the endoplasmic reticulum, whereby the zymogen loses its signal peptide and is *N*-glycosylated at the CHRD level. After removal of the SP, the generated proPCSK9 is autocatalytically cleaved at its internal VFAQ152↓SIP site to form a 13 kDa prodomain and a 62 kDa mature PCSK9 domain, which are noncovalently bound. The proteolytically inactive prodomain-PCSK9 heterodimer, but not its enzymatic activity, is essential for PCSK9 biological function [26]. The protease activity of PCSK9 is interrupted probably to avoid the alignment of the catalytic triad [27] and protect the surrounding proteins from its serine-protease activity [27,28]. 

Of note, when PCSK9 and LDLR co-localize within the secretory pathway of hepatocytes, their interaction leads to LDLR degradation [4,29,30]. Binding of PCSK9 to the ER-resident protein expressed in hepatocytes GRP94, avoids LDLR degradation by preventing early binding of PCSK9 to LDLR [4,29]. Then, the mature PCSK9 is secreted outside the liver cells as an intact heterodimer (62 + 13 kDa) [23].

The half-life of PCSK9 in plasma is about 5 min and one third of circulating PCSK9 is part of LDL particles [31,32] given that it exists in a free and lipoprotein-bound form in the circulation. Furthermore, plasma PCSK9 can be found as an intact heterodimer (62 + 13 kDa), considered the form with a stronger binding to and degradation of LDLR [33], and a furin-cleaved heterodimer (55 + 13 kDa) with reduced affinity for LDLR [34]. 

Unlike other enzymes of the same family [23], the cleaved prosegment of PCSK9 remains bound to the mature enzymatically active protease, retaining it in an enzymatically inactive state. Thus, PCSK9 is the only mature convertase that binds its target without inducing direct cleavage [21]. In individuals with *PCSK9* loss of function (LOF) mutations, the autocatalytic cleavage does not occur. 

## 4. PCSK9 Role on Lipid Homeostasis

**Figure 2:**
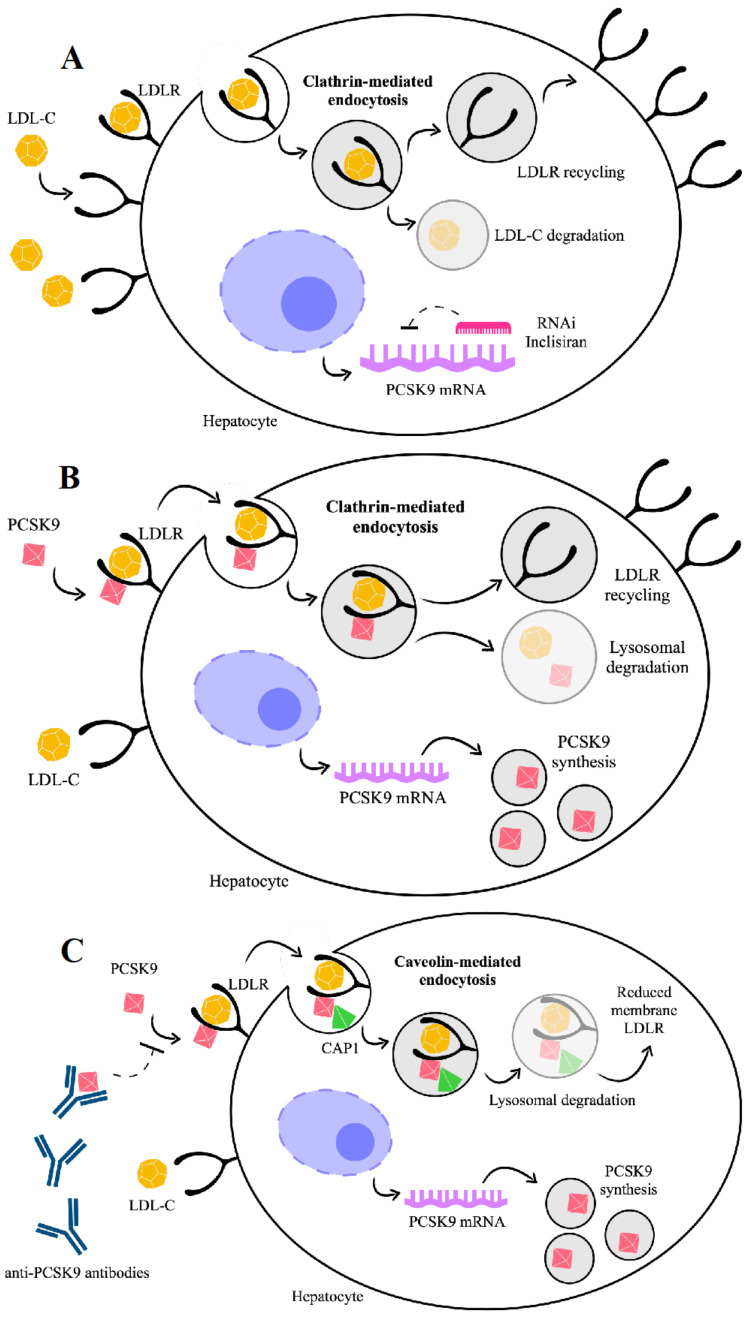
PCSK9 effects on targeting low-density lipoprotein receptor (LDLR) towards lysosomal degradation or recycling, depending on PCSK9 binding to cyclase-associated protein 1 (CAP-1). In the absence of PCSK9 (**A**), the LDLR-LDL complex is internalized into endosomes through a clathrin-dependent mechanism and LDLR is recycled to the cell surface. In the presence of PCSK9, the LDLR-LDL complex is internalized into endosomes through a clathrin-dependent mechanism (**B**) and LDLR is recycled to the cell surface if CAP-1 binding to PCSK9 does not occur. Instead, LDLR undergoes caveolae-dependent endocytosis (**C**) and lysosomal degradation if CAP-1 binds PCSK9. To inhibit PCSK9 action, two approved types of drugs, which are antibody based, act through targeting the extracellular PCSK9 (alirocumab, evolocumab), or through the small interfering RNA, targeting the intracellular synthesis of PCSK9 (inclisiran).

It has been clearly established that PCSK9 plays a crucial role in the regulation of cholesterol homeostasis. To date, the best characterized property of PCSK9 is to promote LDLR degradation by targeting this receptor to the lysosome for degradation [35,36,37] (Figure 2). In turn, LDLR mediates the clearance of PCSK9 from plasma, regardless of the tissue origin of the PCSK9. 

If not bound to PCSK9, LDLR enters the cell while bound to LDL-C, dissociates from LDL-C in the endosomes and is recycled to the cell surface, whereas LDL-C is directed to lysosomes for degradation (Figure 2A). Conversely, if bound to PCSK9, LDLR is internalized and degraded [38] (Figure 2B). PCSK9 binding to LDLR can occur in two phases, a rapid-phase binding with a half-time of 5–10 min and a half-time dissociation of 20 min, and a slow-phase binding with a half-time of ~1.5 h and a half-time dissociation of ~5 h [39]. Based on in vitro and In Vivo experimental models, it has been recently explored in more detail how PCSK9 directs LDLR towards endocytosis and lysosomal degradation after binding to LDLR [40]. A new binding partner for PCSK9, the cyclase-associated protein-1 (CAP-1), has been identified and considered as necessary for the degradation of LDLR by PCSK9. While the PCSK9 catalytic domain binds LDLR, CHRD of PCSK9 seems able to interact with CAP1, leading the protein complex LDLR/PCSK9/CAP1 towards lysosomal degradation through a caveolin-dependent mechanism [40]. In this view, the fate of the LDLR/PCSK9 complex is degradation if dependent on the caveolin pathway or recycling if dependent on the clathrin pathway, and these two different fates depend on the binding partners [40]. 

Reasons advocated for this conclusion are that liver cells cultured with small interfering RNA (siRNA) against *CAP1* as well as in heterozygous *CAP1* knockout mice show the prevention of PCSK9-mediated LDLR degradation. Moreover, LOF polymorphisms in human *PCSK9* (i.e., S668R and G670E) show a defective interaction with CAP1 [40]. 

The multiple cellular pathways by which PCSK9 acts allow us to consider this protein to be able to modulate plasma lipids not solely by affecting hepatic LDLR levels and LDL-C uptake but also by targeting other members of the LDLR family, such as the very low-density lipoprotein (VLDL) receptor, apoE receptor 2 (apoER2) [41] and LDLR-related protein 1 (LRP1) [42]. However, it is possible that the PCSK9 interaction with these receptors does not lead to their degradation, or at least not in all tissues [43].

PCSK9 affects the plasma lipid and lipoprotein levels not only through reduced hepatic lipoprotein clearance but also by promoting hepatic lipogenesis, a phenomenon mediated by both LDLR and apoE, and influenced by transcriptional and post-transcriptional events in hepatic lipogenesis [44]. 

The plasma concentration of LDL cholesterol is the balance among the hepatic secretion of triglyceride-rich VLDL, peripheral conversion of VLDL to LDL and LDL clearance. ApoB-100 is the major protein component of LDL and is essential for LDL particle binding to LDLR. Some evidence has shown that PCSK9 increases apoB-100 secretion in hepatocytes [45] and intestinal cells [46] and decreases VLDLR levels in adipocytes [47], providing a mechanistic basis for an LDLR-independent effect of PCSK9 on cholesterol levels.

Studies on pleiotropic PCSK9 effects assessed in models of *LDLR^−/−^* mice showed that overexpression of human PCSK9 does not affect the expression of genes and proteins involved in hepatic lipogenesis, rather it increases the intestinal source of plasma cholesterol and triglycerides. These findings were suggestive for LDLR-independent effects of PCSK9 on triglyceride-rich lipoprotein secretion and specific to enterocytes [46,48]. Thus, PCSK9 increases intestinal triglyceride-rich lipoprotein production through both LDLR-dependent and -independent mechanisms [48].

PCSK9 activity induces the degradation of LDLR and, in an LDLR- and catalytic activity-independent manner, of its closest structural members, such as VLDL-R and ApoER2 [41], because of the presence, in these two receptors, of an epidermal growth factor-like repeat A (EGF-A) domain similar to that of LDLR [49]. The catalytic subunit of PCSK9 binds to the LDLR EGF-A domain, and the complex is internalized and targeted to the lysosomes for destruction. 

At least 250 mutations have been identified so far, distributed in all PCSK9 domains [11]. *PCSK9* gene variants are the cause of only 2–4% of ADH; nevertheless, they influence the circulating cholesterol concentration in the general population much more than *LDLR* or *APOB* polymorphisms, the other two genes responsible, respectively, of 85–90% and 1–12% of familial hypercholesterolemia [50,51,52].

Even though PCSK9 interacts with LDLR primarily via its catalytic domain, the majority of human PCSK9 mutations are distributed in the SP (*n*  =  7; 10%), prodomain (*n*  =  21; 28%) or CHRD (*n*  =  22; 30%) [11].

**Figure 3:**
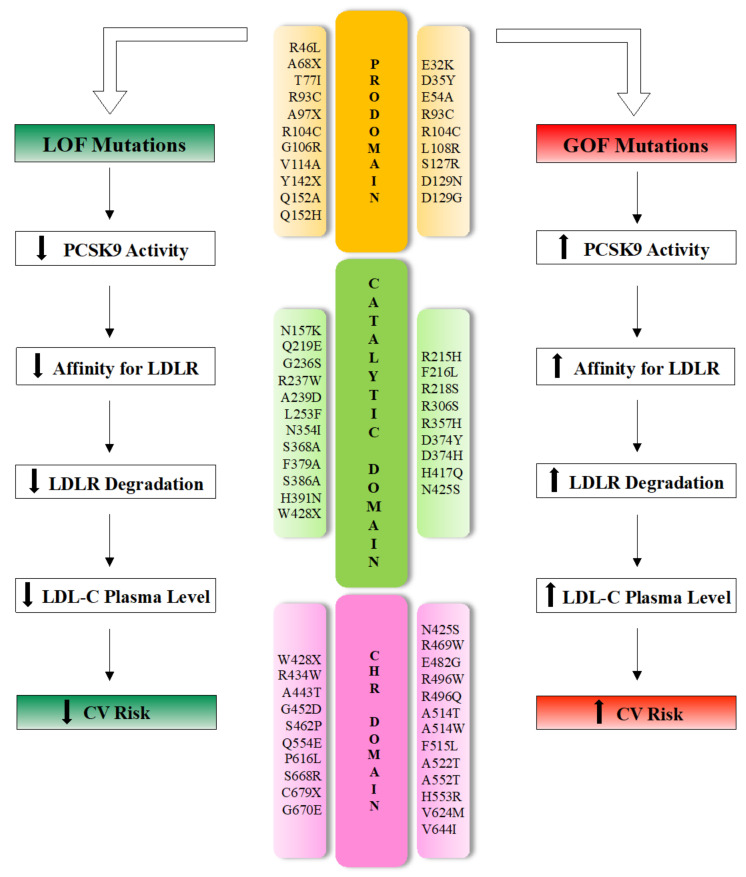
Effects of the main natural mutations of the PCSK9 domains on cholesterol homeostasis phenotype and their impact on cardiovascular disease risk. LOF: loss-of-function; GOF: gain-of-function; LDL: low-density lipoprotein; LDLR: LDL receptor; LDL-C: LDL cholesterol; CV: cardiovascular; CHR: cysteine- and histidine-rich domain.

With respect to the functional outcome on CV risk, *PCSK9* sequence variants produce mild to moderate (and opposing) phenotypes, classified as GOF or LOF mutations (Figure 3).

Patients with GOF mutations are prone to increased LDL-C levels and develop familiar hypercholesterolemia accompanied by increased CV risk [53]. Experimental data derived from cell transfection and animal model experiments demonstrated that overexpression of wild-type or mutant *PCSK9* induces a marked reduction in hepatic LDLR protein and hypercholesterolemia [22,43,54]. In contrast, mice lacking the *PCSK9* gene *(*pcsk9 ^−/−^*)* show increased LDLR molecule expression in the liver, increased removal of circulating LDL and reduced plasma LDL-C levels [55].

In humans, GOF mutations in *PCSK9*, such as the substitution of Asp with Tyr (PCSK9-D374Y), can cause severe phenotypes with cholesterol levels of >500 mg/dL (∼13 mmol/L) [56,57].

On the other hand, carriers of LOF mutations in *PCSK9* show a higher density of liver LDLR, reduced LDL-C levels and a lifetime risk of cardiovascular disease reduced by between 50 and 86% compared with non-carriers [13,58,59]. Polymorphisms leading to LOF in the *PCSK9* gene are relatively common and have been gradually established both in in vitro and In Vivo studies [40,55].

Several LOF mutations in *PCSK9*, leading to low plasma PCSK9 levels and consequent hypocholesterolemia, are the cause of impaired processing (e.g., S386A), trafficking (e.g., R46L) or secretion (e.g., S462P) [60,61,62].

PCSK9 has also been proposed to modulate lipoprotein (Lp) (a) via the LDLR pathway with data showing a significant increase in plasma Lp(a) concentrations in patients with *PCSK9* GOF mutations [63].

## 5. PCSK9 Effects on Atherosclerosis

Atherosclerotic plaque formation is a lipoprotein-driven disease characterized by intimal inflammation, proliferation, necrosis and calcification in particular sites of the arterial tree [64]. While the major sources of circulating PCSK9 are the liver, kidney and small intestine [65], PCSK9 is also produced by vascular cells such as vascular smooth muscle cells (VSMC), endothelial cells (EC) and, at lower level, macrophages [66,67,68]. PCSK9 secreted by VSMC, which express more PCSK9 than EC [69], acts in a paracrine manner, downregulating LDLR expression on the cell surface of macrophages [66] and avoiding the formation of foam cells. These findings can induce to suppose that PCSK9-stimulated macrophages may reduce foam cell formation and hence atherosclerosis progression [66]. However, native LDL molecules are not the major source of cholesterol accumulation in macrophages. Macrophages are able to oxidatively modify accumulated LDL to form aggregated and oxidized-LDL (oxLDL), the major source of cholesterol ester accumulation in macrophages and VSMC [70,71,72]. Endocytosis of oxLDL particles in monocytes and macrophages does not require LDLR but scavenger receptor (SR) A, cluster of differentiation 36 (CD36) and lectin-like oxidized low-density lipoprotein receptor 1 (LOX-1), whose expression is highly increased under different inflammatory stimuli [73,74,75], thus leading to oxLDL accumulation in arterial walls [76,77]. Unlike native LDL uptake through LDLR, SR uptake is not subject to feedback inhibition by intracellular sterols, and receptor uptake can continue unabated so long as modified LDL is in the extracellular milieu [78]. Moreover, the inflammatory milieu in vascular tissue potentiates the cross-talk between PCSK9 and LOX-1, in which PCSK9 stimulates LOX-1 and LOX-1 stimulates PCSK9 [67].

Experimental evidence shows that PCSK9 increases both gene and protein levels of SRA, CD36 and LOX-1, leading to increased oxLDL uptake. If macrophages lack these receptors, this increased lipid uptake is abolished, suggesting that all three receptors are involved in oxLDL uptake and in the formation of foam cells [74].

In line with these observations, macrophages transfected with PCSK9 siRNA show lower CD36 mRNA expression and lower intracellular cholesterol accumulation than the control group [74]. 

OxLDL entry into macrophages is through SR CD36. The accumulation of cholesterol crystals, mainly due to the imbalance between esterified and free cholesterol, promotes the activation of the innate immune signaling complex nucleotide-binding and oligomerization domain (NOD)-like receptor pyrine domain-containing protein 3 (NLRP3) inflammasome [79], the key mediator of interleukin (IL)-1 family cytokine production in atherosclerosis. Consequently, NLRP3 inflammasome activation is deeply involved in the vascular inflammatory response driving development and progression of atherosclerosis. Reduction of cholesterolemia reduces cholesterol crystal formation and atheromas [80].

Indeed, PCSK9 promotes atherogenesis both indirectly, by raising plasma lipids, and directly via modulation of EC apoptosis [81], with a consequent reduction in vessel stability, as well as expression of adhesion molecules, chemoattractants and inflammatory cytokines that amplify inflammation at the atherosclerotic site [82]. PCSK9 shows a pro-inflammatory behavior, upregulated in macrophages pro-inflammatory cytokines and chemokine genes, e.g., IL-1β, *IL-6*, *tumor necrosis factor (TNF)-α*, *CXCL2* and *monocyte chemoattractant protein* (*MCP)-1* [83]. Accordingly, in an atherosclerosis-prone mouse model, the deletion of the *PCSK9* gene, independently of LDLR, reduced the expression of adhesion molecules from EC, such as ICAM-1, and chemotactic factors, e.g., MCP-1 and MCP-3, all promoting monocyte adhesion and infiltration into the vessel wall [84].

It is known that shear stress is a critical component for atherosclerosis development and progression [85]. Both EC and VSMC show PCSK9 protein expression is higher under low blood flow than high blood flow, an effect potentiated by lipopolysaccharide (LPS) stimulation [69,86]. Therefore, a negative correlation exists between PCSK9 vascular expression levels and blood flow. 

The key role of PCSK9 in the atherosclerotic process is also confirmed by a In Vivo experimental model conceived to obtain a quick and convenient model to study atherosclerosis in knockout or transgenic mice without having to generate double knockout on an *ApoE*^−/−^ or *LDLR*^−/−^ background [87]. In particular, a single injection of a GOF murine *PCSK9* mutant plasmid (pAAV/D377Y-mPCSK9) into C57 mice to effectively knockdown *LDLR* resulted in significant hypercholesterolemia and subsequent atherosclerosis development within 3 months without the need of germline knockout of *ApoE* or *LDLR* [88]. A single AAV9-PCSK9 injection, which reduced LDLR expression by more than 90%, and a high-fat diet induced a significant hypercholesterolemia (total cholesterol ~700 mg/dL) from 1 week and up to 3 months. 

In addition, the proatherogenic role of PCSK9 is supported by evidence showing a positive association between PCSK9 and local vessel stiffness [89], and the significant improvement of endothelial function and arterial stiffness after a short-term treatment with PCSK9 inhibitors [90,91].

These findings support the concept that, in addition to its well-established action on LDLR modulation in hepatocytes, PCSK9 can also exert direct effects on atherogenesis in the absence of systemic lipid changes, engaging with other receptors or proteins involved in atherosclerosis pathogenesis [92,93]. Indeed, in a 15-year prospective cohort study, serum PCSK9 concentrations predicted CV events even after adjustments for established CVD risk factors, including LDL-C [94]. In another study, PCSK9 levels were significantly associated with 10-year progression of carotid plaque, independently of LDL-C levels [95]. 

PCSK9 can affect the content and size of atherosclerotic lesions [96]. PCSK9 accumulation in the lesion directly affects plaque composition, also independently of serum lipid levels. This fact could explain the direct relationship between PCSK9 and atherosclerosis and why PCSK9 overexpression is proatherogenic, whereas its absence is protective, adding cardiovascular benefits for anti-PCSK9 therapies.

## 6. PCSK9 and Thrombosis 

The atherothrombotic process underlies acute coronary and cerebrovascular events where the activation of inflammatory mechanisms is strictly dependent on interaction among different cell types, such as platelets, leukocytes and cells of the vascular wall. Indeed, factors other than the mere presence of an atherosclerotic lesion need to be involved for CV events and in this scenario the thrombogenic potential of the circulating blood may play a key role [97]. There is a significant association between high cholesterol levels and thrombosis caused by plaque rupture (versus erosion) [98,99].

Mice with a PCSK9 deficiency appear to have a similar phenotype to PCSK9-deficient humans with low levels of LDL-C and protection from atherosclerosis. Lipid reduction attenuates chronic inflammatory responses in arterial disease [55,96] and has beneficial effects on inflammatory responses in venous disease [100]. In comparison with wild-type animals, mice with PCSK9 deficiency developed less venous thrombosis caused by inferior vena cava (IVC) ligation [101], given that IVC thrombosis was evident in 60% of wild-type mice and 25% of *pcsk9* ^−/−^ mice. Furthermore, when formalin fixed thrombi were analyzed, the cellular composition analysis revealed a greater weight and size of the thrombus, more myeloid cell recruitment and neutrophil extracellular trap formation (NETs) in wild-type compared to *pcsk9* ^−/−^ mice [100]. In response to inflammatory stimuli, neutrophils generate NETs, meshworks of DNA fibers comprising histones and antimicrobial proteins that disarm and kill pathogens [102]. NETs interact closely with fibrin strands in the thrombus, thus potentially influencing thrombus organization and stability. Furthermore, histones in NETs or liberated after digestion of NET-DNA could also provide a stimulus for platelet aggregation [103,104] and activation of the coagulation cascade. Deficiency in PCSK9 is associated with protection from thrombosis and reduced leukocyte recruitment and NET formation at the site of thrombosis [100]. In this way, theoretically, PCSK9 inhibition should have an antithrombotic effect even though PCSK9 inhibitors, differently from statins, show no effect on fibrinogen and D-dimer levels in statin-intolerant patients with familial hypercholesterolemia [105]. 

**Figure 4:**
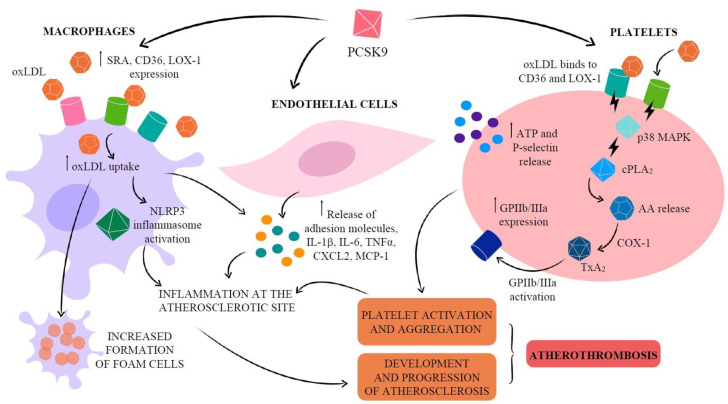
Molecular pathways implicated in the PCSK9 effects on endothelial cells, macrophages and platelets, leading to atherothrombosis. LDL: low-density lipoprotein; oxLDL: oxidized LDL; LOX-1: lectin-like oxidized low-density lipoprotein receptor 1; SRA: scavenger receptor A; CD36: cluster of differentiation 36; NLRP3: NOD-like receptor pyrine domain-containing protein 3; PLA_2_: phospholipase A_2_; AA: arachidonic acid; COX: cyclooxygenase; TX: thromboxane; GPIIb/IIIa: glycoprotein IIb/IIIa.

**PCSK9 and platelets**: Thrombosis strictly depends on the adhesion, activation and aggregation of platelets [106]. The association between hypercholesterolemia and prothrombotic propensity due to increased platelet biogenesis, turnover and activity is not new [107]. Platelets have long-established roles central to hemostasis initiation and thrombus formation [108] and are increasingly recognized to be versatile effector cells linking thrombosis and inflammation. Activated platelets interact with endothelial cells of inflamed or atherosclerotic arteries and release platelet factors that trigger an inflammatory reaction of endothelial cells and/or facilitate leukocyte—endothelial interactions [109,110]. In the presence of intact vascular endothelium, the release of prostacyclin (PGI_2_) and nitric oxide (NO), two major anti-aggregants, regulates the balance between pro- and anti-aggregants and prevents the formation of a thrombus inside the blood vessel [111]. However, in subjects at risk of arterial thrombosis, this key protective pathway is overcome, and the alteration of platelets, as well as altered vascular reactivity, may contribute to abnormal vascular responses in atherosclerosis. The loss of PCSK9 in experimental animal models has suggested a role for PCSK9 in platelet reactivity also because a significant reduction in the expression of the platelet activation markers glycoprotein IIb/IIIa and P-selectin was found in *PCSK9* knockout mice in comparison with wild-type ones [112]. A positive correlation between circulating PCSK9 levels and platelet markers [112,113,114,115] has been documented. The ability of platelets to adhere to the uncovered thrombogenic subendothelial matrix of the ruptured atherosclerotic plaque with their subsequent activation and aggregation strongly increases the risk of ischemic events [116]. As a result, thrombosis may be promoted in patients with ACS undergoing percutaneous coronary intervention (PCI), thus justifying the treatment with antiaggregant agents. In a study carried out on patients with a recent ACS event undergoing PCI, serum PCSK9 levels were independently correlated with high-on-treatment platelet reactivity and a higher incidence of atherothrombotic outcomes [116]. Indeed, the relationship between hypercholesterolemia and platelet activation in patients with cardiovascular disease is known [109,117]. The increased levels of PCSK9 in these patients could indirectly activate platelets for the impaired clearance of lipoproteins, which perform important regulatory effects on platelets by native LDL and oxLDL (Figure 4). 

On the other hand, PCSK9 plays an intrinsic role in promoting platelet activation since PCSK9 has been shown to significantly enhance platelet aggregation and expression of integrin αIIbβ3 [112], and directly induce platelet release of ATP and P-selectin from granules [118]. In particular, PCSK9, through the CD36 and LOX-1 downstream activation of Src kinase and mitogen-activated protein kinase (MAPK)-extracellular signal-regulated kinase 5 and c-Jun N-terminal kinase pathways, leads to increased cyclooxygenase-1 (COX-1) activity and, consequently, thromboxane A_2_ synthesis [118]. Moreover, these PCSK9 effects on platelet activation are inhibited by aspirin [118], thus supporting the potential benefit in preventing platelet hyperreactivity if administration of this anti-aggregant agent is done in addition to anti-PCSK9 therapy in clinical settings characterized by higher levels of PCSK9. In a cross-sectional study on stable coronary disease patients, a positive and independent association was found between plasma PCSK9 levels and platelet count [119], a reliable marker for various diseases, associated with morbidity, pathophysiology and mortality due to coronary disease [120,121]. 

Atrial fibrillation has been reported to be a condition of higher PCSK9 concentrations linked to increased platelet activation. Actually, a positive correlation was found between PCSK9 and urinary 11-dehydro-thromboxane B_2_ excretion, a metabolite predominantly, but not exclusively, derived from platelets. This finding could reflect either platelet COX-1-dependent thromboxane generation or COX-2-dependent biosynthesis by inflammatory cells and/or platelets in clinical settings characterized by low-grade inflammation [122].

The study on influence of anti-PCSK9 monoclonal antibody (mAb) treatment on platelets from subjects affected by familial hypercholesterolemia was first carried out in our laboratory. We demonstrated a reduction in platelet aggregability and activation after a treatment for 2 up to 12 months with the anti-PCSK-9 mAbs alirocumab or evolocumab. This improvement was evident in the presence of concomitant therapy with aspirin suggesting an effective role of treatment with PCSK9-inhibitors in increasing the sensitivity to the antiplatelet effects of aspirin [114]. Positive correlations were found not only between PCSK9 and LDL-C but also PCSK9 levels and the platelet activation markers soluble CD40L, Platelet Factor-4 and P-Selectin. 

Although, as aforementioned, the direct effect of PCSK9 in promoting platelet aggregation and activation has been established, the reduction of platelet hyperreactivity due to PCSK-9 inhibitors is supposed to be mainly related to cholesterol reduction. As known, cholesterol accumulation in plasma membranes alters the membrane structures with effects on the signaling of the surface receptors. In its native form, LDL alone does not induce platelet aggregation but increases platelet response to pro-aggregant agents; if oxidized, LDL particles stimulate platelet aggregation also in the absence of agonists [123]. The oxLDL-mediated generation of reactive oxygen species (ROS) is one of the mechanisms involved in the reduced NO bioavailability at all stages of atherosclerosis [124,125], and a critical determinant of platelet function. Therefore, a hypercholesterolemia state may be characterized by platelet hyperactivity also for the hyporesponsiveness to NO-related pathways [126,127,128,129,130]. 

**Figure 5:**
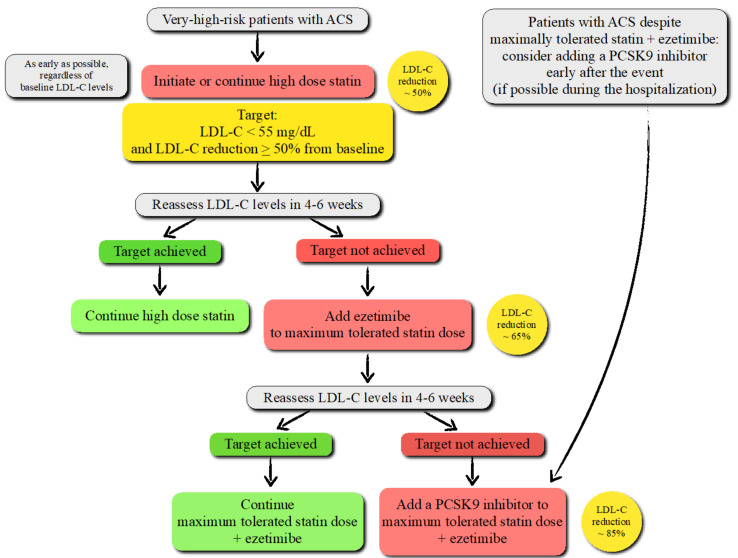
Treatment algorithm to pharmacologically decrease low-density lipoprotein cholesterol (LDL-C) levels in patients with acute coronary syndrome (ACS) according to the recent European Society of Cardiology (ESC)/European Atherosclerosis Society (EAS) guidelines.

**PCSK9 and ACS**: While the American College of Cardiology and American Heart Association guidelines recommend a more intensive lipid-lowering therapy for LDL-C ≥ 70 mg/dL [131], the European Society of Cardiology/European Atherosclerosis Society (EAS) guidelines recommend the addition of ezetimibe and, as a further step, anti-PCSK9 mAbs to high-intensity statins if patients with ACS fail to achieve an LDL-C reduction by ≥ 50% from baseline and LDL-C < 55 mg/dL [132] (Figure 5).

Patients who have experienced an ACS are at very high risk of recurrent atherosclerotic CV events. The new LDL-C goal (< 55 mg/dL), based on robust evidence on safety and efficacy emerged from several randomized controlled clinical trials and meta-analyses, is associated with a significant reduction in post-ACS CV events after the addition of ezetimibe and PCSK9 inhibitors to background statin therapy [133,134,135]. Further intensification with anti-PCSK9 mAbs is also recommended for high-risk patients experiencing an ACS despite being on the treatment with maximally tolerated statins and ezetimibe [131,132]. 

PCSK9 inhibitors have been shown to decrease the risk of major adverse CV events more than statins despite the same LDL-C reduction [2]. The ability of anti-PCSK9 mAb therapy to improve CV outcomes in ACS patients may be due to inhibition of direct PCSK9 effects exerted on endothelial function, plaque stabilization and platelet activation, and indirectly to its dramatic effects on lipid homeostasis [136,137]. 

Plasma levels of PCSK9 have been reported to be increased in patients hospitalized for ACS in a prospective study where coronary lesions were assessed using SYNTAX scores. Serum PCSK9 concentrations measured on admission for ACS and on every day of hospitalization were positively associated with SYNTAX scores even after adjusting for LDL-C and the major CV risk factors [138].

**PCSK9 and Sepsis**: PCSK9 overexpression exacerbates the hypercoagulable and pro-inflammatory states in early sepsis, a complex disease characterized by organ dysfunction along with systemic activation of inflammation and coagulation, as demonstrated in experimental models of mice overexpressing PCSK9 [47,139] and findings on humans [140]. *PCSK9* LOF mutations improve survival outcomes during sepsis, while *PCSK9* GOF mutations increase mortality and thus are detrimental in septic shock patients. Mice overexpressing PCSK9 show increased levels of thrombin–antithrombin (TAT) complexes, thereby contributing to a hypercoagulable state in sepsis. Conversely, *PCSK9* deficiency reduces circulating levels of cell-free DNA (cfDNA), a strong prognostic biomarker in sepsis [102,141,142] and considered as an important link among innate immunity, inflammation and coagulation, with both procoagulant [143,144] and antifibrinolytic [145] properties. cfDNA triggers the intrinsic pathway of blood coagulation [143,144,146] and elevated levels of cfDNA are found in patients with deep venous thrombosis [101,142]. Indeed, cfDNA circulates at low levels in healthy individuals, with elevated levels observed in an array of clinical conditions, including myocardial infarction [147] and sepsis [148]. cfDNA increases plasma thrombin generation in the absence of platelets, through factor XI- and factor XII-dependent mechanisms, and the ability of cfDNA to integrate into fibrin clots has been shown to inhibit plasmin-mediated fibrinolysis. Therefore, it has been hypothesized that, in sepsis, the reduction of cfDNA in *PCSK9* knockout mice could reduce coagulopathy. Actually, complete *PCSK9* knockout seems to be protective in sepsis [139]. 

## 7. Current Drugs to Inhibit PCSK9

Although statin therapy remains the gold standard for reducing cholesterol and circulating LDL-C in patients with high risk for CVD, monotherapy with statins has not been optimized in clinical practice given that over 70% of patients affected by clinical atherosclerotic CVD do not reach LDL-C < 70 mg/dL levels [149]. Moreover, the need of a daily statin administration and the appearance of real or perceived adverse effects, such as myopathies and myalgias, induce patients to discontinue drug intake within 1 year. Noteworthy, poor adherence to therapy is a major factor involved in the increased risk of CVD [150].

Therefore, the need for new well-tolerated therapies for lowering LDL-C and strategies to improve the adherence of patients to currently available therapies induced pharmaceutical industries to open a new era for lipid-lowering therapy.

Soon after the discovery of PCSK9 as a molecule with a function in lipid metabolism, there was a surge in interest to develop therapies to target its pathway as a treatment for hypercholesterolemia. 

Bearing in mind that the majority of LDLR are reused, and PCSK9 acts to prevent its intracellular recycling, the block with a monoclonal antibody will result in promoting the recycling of LDLR on cell membranes, thereby inducing the uptake of LDL-C from plasma and a reduction in the circulating LDL-C levels [151,152,153]. Thus far, the two fully human mAbs, alirocumab and evolocumab, developed by transgenic mice platforms, prevent the interaction between PCSK9 and EGF-A on LDLR. Alirocumab and evolocumab have been approved for treating hypercholesterolemia in the clinic as an adjunctive therapy to the standard of care for patients with established CVD and/or familial hypercholesterolemia [154]. These anti-PCSK9 mAbs are able to markedly decrease circulating LDL-C levels up to 60–70%, followed by significant effects on the reduction of cardiovascular risk [155,156]. 

The Further Cardiovascular Outcomes Research with PCSK9 Inhibition in Subjects with Elevated Risk (FOURIER) is a randomized, double-blind, placebo-controlled, multinational clinical trial involving 27,564 patients randomized from February 2013 to June 2015 at 1242 sites in 49 countries. The aim of this trial was to test the clinical efficacy and safety of evolocumab when added to high-intensity or moderate-intensity statin therapy in patients with clinically evident atherosclerotic CVD. The median duration of follow-up was 26 months and the study showed that the percentage reduction in LDL-C levels with evolocumab (either 140 mg every 2 weeks or 420 mg every month) was 60%, from a median baseline value of 92 mg/dL to 30 mg/dL [155]. In addition, evolocumab treatment significantly reduced by 15% the risk of the primary end point (major cardiovascular events, MACE) [155], with benefits also in patients with diabetes [157], metabolic syndrome [158] or prior ischemic stroke [159]. Moreover, from the whole treated group, 0.3% of patients exhibited the production of binding antibodies against evolocumab, but not neutralizing.

The ODYSSEY OUTCOMES study is a multicenter, randomized, double-blind, placebo-controlled trial involving 18,924 patients that undergone randomization from November 2012 through November 2015 at 1315 sites in 57 countries. Its aim was to test the clinical efficacy and safety of alirocumab (75 to 150 mg every 2 weeks adjusted under blinded conditions) when added to the maximally tolerated high-intensity or moderate-intensity statin therapy in patients with coronary syndrome 1 to 12 months earlier and levels of high atherogenic lipoproteins despite current statin therapy. Patients were followed for a median of 2.8 years and the study demonstrated the superiority of alirocumab vs. the placebo in LDL-C lowering (>50% LDL-C reduction) and cardiovascular outcome (reduction by 15% of MACE) (i.e., first occurrence of coronary heart disease (CHD) death, non-fatal myocardial infarction, fatal/nonfatal stroke and unstable angina pectoris, requiring admission to the hospital) [156]. Similarly, both trials showed that subjects with a larger absolute and relative risk reduction were those with a high risk score [160,161], and both evolocumab and alirocumab are able to reduce by 31% the risk of venous thromboembolism [162].

Both trials, FOURIER and ODISSEY OUTCOMES, supported the role of a PCSK9 inhibitor as an adjunctive strategy for the treatment of dyslipidemia, as established by clinical practice guidelines of the American College of Cardiology [154], which recommend the administration of PCSK9 inhibitors for patients with established clinical atherosclerotic CVD and LDL-C ≥ 90 mg/dL, despite the use of maximally tolerated statin therapy and/or ezetimibe.

The need for biweekly or monthly subcutaneous injections has generated concerns for patient compliance and a risk of low compliance has been noted. Indeed, a small retrospective analysis conducted in a real-world setting revealed that adherence rates to anti-PCSK9 mAbs appear to be better than those to statins [163]. This observation, at least in part, is also due the sense of reassurance for the substantial reduction in LDL-C and the achievement of recommended LDL-C targets (even in the case of very high starting levels) [164]. Of course, a greater importance was attributed to an injection every 2–4 weeks versus an oral daily therapeutic regimen [164]. However, among patients, about 12% were only partially adherent and 9% nonadherent [165]. 

Since the infrequent administration regimen can increase the number of patients who maintain their therapeutic goals, a new pathway based on RNA interference therapy to inhibit the cellular production of PCSK9 has been investigated. Actually, the new anti-PCSK9 inclisiran sodium (inclisiran) significantly reduces hepatic production of PCSK9, with a consequent marked reduction in LDL-C levels, and, importantly, preserves its pharmacodynamic effects when administrated subcutaneously every 6 months. In light of this, inclisiran’s twice-yearly dosing would represent an advantage over the biweekly or monthly dosing for PCSK9 mAbs, considering that LDL-C reductions produced by inclisiran are substantial and similar to those of high-intensity statins (>50% LDL-C reduction), adding an important increase in adherence and treatment compliance. Therefore, inclisiran has the potential to be used for the primary and secondary prevention of cardiovascular disease. 

Inclisiran is a chemically synthesized siRNA targeting PCSK9. After 24 h from its injection, inclisiran is undetectable in plasma because of the highly specific and rapid uptake by the hepatocyte [166]. This suggests that even though PCSK9 is present in extrahepatic tissues, at least to date, no off-target effects for inclisiran have been detected.

The guide strand of this small siRNA binds intracellularly to an RNA-induced silencing complex (RISC), which hybridizes to the mRNA molecules encoding PCSK9 specifically, cleaving the mRNA. The cleaved mRNA is degraded catalytically to prevent PCSK9 protein synthesis in the liver, thereby one inclisiran–RISC complex can degrade multiple PCSK9 mRNAs [167]. 

The LDL-C lowering efficacy and clinical safety of inclisiran have been assessed within the ORION trial program. Ongoing Phase III trials will provide evidence on longer-term safety and effectiveness, and on inclisiran’s efficacy in patients with homozygous familial hypercholesterolemia. 

All three phases III ORION studies (i.e., ORION-9, ORION-10 and ORION-11) have confirmed the efficacy of inclisiran (284 mg) in the long-term reduction of LDL-C with reduced plasma PCSK9 levels by approximately 80% in patients at very high CV risk, along with its tolerability [168]. 

Furthermore, the ongoing multinational ORION-4 and ORION-5 trials are assessing inclisiran’s impact on cardiovascular outcomes, respectively, in adults with established atherosclerotic CVD and in adults with homozygous familial hypercholesterolemia [169].

Collectively, the encouraging data from phase III pivotal studies have contributed to the EU’s December 2020 approval of inclisiran as a treatment for primary hypercholesterolemia (heterozygous familial and non-familial) or mixed dyslipidemia [169]. Noteworthy, a different approach to inhibit PCSK9 synthesis may originate from base editors applied to make precise single-nucleotide changes. Actually, new gene-editing technologies are becoming promising tools for future therapeutic applications, also in the management of hypercholesterolemia by targeting PCSK9 [170,171].

## 8. Conclusions

Unknown almost 20 years ago, soon after its discovery and still now PCSK9 remains common parlance among specialists focused on prevention and treatment of atherosclerotic CVD.

The association between some genetic variants of *PCSK9* and plasma LDL-C levels in humans is very strong, being associated either with hyper- or hypo-cholesterolemia. Studies of in vitro and In Vivo animal models have provided consistent insight into the role of PCSK9 on LDL metabolism regulation and its association with CVD, subsequently translated into an effective medical therapy in record time. 

PCSK9’s impacts on atherosclerosis progression has been proved by the benefits observed in patients who followed PCSK9-targeted therapies. Indeed, beyond its effects on LDL metabolism, several studies have demonstrated the existence of additional roles of PCSK9 in different stages of atherosclerosis through its interaction with other receptors, such as LRP1, ApoER2, and CD36. PCSK9 derived from the plasma, as well as VMSCs and macrophages, may contribute to the development of atherosclerotic plaque. Furthermore, PCSK9 has been shown to modulate thrombosis by promoting platelet activation, leukocyte recruitment and clot formation.

These findings have been translated into newly approved therapeutic approaches, evolocumab and alirocumab, which by inhibiting PCSK9 activity are able to dramatically reduce the LDL-C levels and attenuate atherosclerotic cardiovascular events. Lastly, an additional therapeutic PCSK9 inhibitor, inclisiran, compared with PCSK9 mAbs, has as its main benefit a less frequent dosing regime.

## Data Availability

Not applicable.
